# Knowledge, Attitude, and Practice Related to Child Abuse Among Doctors in Mangalore, India: A Cross-Sectional Study

**DOI:** 10.7759/cureus.88919

**Published:** 2025-07-28

**Authors:** Neil Dsouza, Sandria A Dsouza, Melisha G Dsouza, Jennifer A, Jason G Dsouza

**Affiliations:** 1 Pediatrics, Father Muller Medical College Hospital, Mangalore, IND

**Keywords:** attitude, child abuse, doctors, knowledge, practice, reporting

## Abstract

Introduction: Abuse can have serious implications on children, persisting even into adulthood. The prevention of child abuse is therefore paramount, as it can present in the form of sexual, psychological, and physical abuse in addition to neglect.

Objectives: To assess the knowledge, attitude, and practice (KAP) of doctors toward child abuse in the region of Mangalore, India, using a validated self-administered questionnaire. To compare the level of knowledge and attitude between the various professional groups.

Materials and methods: A descriptive cross-sectional study was conducted among allopathic doctors, including interns, postgraduate students, and staff doctors, using a validated self-administered questionnaire. The study was conducted over three months (May-July 2024) using a convenience sampling method, with data being collected via Google Forms (Google LLC, Mountain View, CA, USA) and printed questionnaires. Informed consent, along with a participant information sheet containing brief details of the study, was provided prior to inclusion. Statistical analysis performed included descriptive statistics, Mann-Whitney U tests, and Kruskal-Wallis tests.

Results: A total of 317 doctors participated. The mean knowledge score was 10.03 (SD ± 1.66) (Max score - 14), with 57.1% (n = 181) having adequate knowledge, 40.1% (n = 127) having good knowledge, and 2.8% (n = 9) obtaining poor knowledge scores. Higher knowledge scores were significantly associated with age (p = 0.016), gender (p = 0.001), and years of practice (p = 0.021). The mean attitude score was 4.23 (SD ± 1.10) (Max score - 6), with no significant differences across professional and demographic characteristics (p > 0.05). 99.7% (n = 316) of doctors agreed it was important to raise awareness on child abuse among the medical fraternity. Despite adequate knowledge and attitude, the practice of doctors towards reporting of abuse was unsatisfactory.

Conclusion: While knowledge and attitude towards child abuse were found to be adequate and moderate, respectively, significant gaps exist in translating knowledge into practice. Strengthening the medical curriculum and implementing targeted training programs could improve child abuse detection and reporting among doctors.

## Introduction

The abuse of children, once thought to be an issue of the past, now presents itself as an epidemic plaguing countries worldwide, affecting even developed nations. The World Health Organization (WHO) has defined child maltreatment as “all forms of physical and emotional ill-treatment, sexual abuse, neglect, and exploitation that results in actual or potential harm to the child’s health, development or dignity,” affecting children under the age of 18 years [1]. A nationwide study in India in 2007 reported that two out of every three children had experienced physical abuse, with 72.7% of children aged 5-12 years being victims [2]. The consequences of abuse on children can have lasting negative effects on mental health, and the effects can be immediate, intermediate, and long-term. In adolescence, it has a strong association with hopelessness, suicidal ideation, and attempting suicide [3]. Children tend to stay silent and do not reveal the truth. Abuse triggers strong feelings of fear, uncertainty, shame, guilt, rage, helplessness, depression, and distress in them. These children may perceive themselves as different: disgusting and damaged [4]. The criteria for post-traumatic stress disorder diagnosis are met by 30-40% of preadolescent children who are abused [5]. 

Children are a nation's most valuable resource, and it is of utmost importance to safeguard our children to ensure a better future for the country. Healthcare professionals play a critical role in identifying and managing child abuse. However, studies indicate a lack of awareness and insufficient reporting practices among doctors. Hence, this study is being undertaken to assess knowledge, attitudes, and practices (KAP) of doctors regarding child abuse in the region of Mangalore, India. By gaining insight into these aspects, the study hopes to contribute to the development of more effective strategies for the early recognition and management of child abuse in Indian medical practice.

## Materials and methods

Study design

This descriptive cross-sectional study was conducted over three months (May-July 2024) among allopathic doctors practicing within the city limits of Mangalore, Karnataka, India. A convenience sampling method was used to collect the data.

Inclusion Criteria

Allopathic doctors, including interns and postgraduates from various medical colleges currently practicing in the region of Mangalore. Private medical practitioners were also included.

Exclusion Criteria

MBBS students (first year to final year) and other healthcare professionals (veterinary, Ayurveda, homeopathy, physiotherapy, nursing).

Instrument development

The questionnaire was developed from available literature and in-depth discussions with subject experts [6-8]. The questionnaire utilized is included in the Appendices. Content validation was performed by two consultants in the fields of community medicine and forensic medicine. It was in English and consisted of 33 questions divided into four categories: (i) Section A: Demographic characteristics of participants (6 questions); (ii) Section B: Knowledge (14 questions); (iii) Section C: Attitude (7 questions); and (iv) Section D: Practice (6 questions).

Scoring and categorization

Each correct response was awarded a score of 1, with a maximum score of 14 and 6 in the knowledge and attitude categories, respectively. Responses in the practice section were analyzed descriptively along with a single attitude question. An arbitrary scale using <50%, 50 - 75%, and >75% was used to stratify responses into the following categories: (i) Knowledge: Poor (0 - 6), Adequate (7 - 10), Good (11 - 14); (ii) Attitude: Poor (0 - 2), Moderate (2-4), Positive (4-6).

Data collection

The questionnaires were distributed either online (Google Forms, Google LLC, Mountain View, CA, USA) or as physical copies among eligible doctors. Respondents were encouraged to complete the survey upon receipt. Informed consent was taken prior to inclusion in the study. Responses generated were tabulated in a Microsoft Excel 2016 spreadsheet (Microsoft Corp., Redmond, WA, USA).

Sample size estimation

Sample size estimation was based on a pilot study conducted with a group of 30 participants (10 each from interns, postgraduates, and staff). Using the formula depicted below, the sample size was calculated as follows:

$n =\frac{Z_{\alpha}^2 \cdot p \cdot (1 - p)}{d^2}$

where Z_α_​ = 1.96 at a 95% confidence interval, p = 73.3% (n = 22) had good knowledge regarding child abuse, and e = 5%. Therefore, the total sample size = 301.

Ethical approval

Ethical approval was granted by the Father Muller Institutional Ethics Committee (FMIEC), Father Muller Research Centre, Mangalore, with reference number FMIEC/CCM/346/2024. The data was anonymous with no identifiers collected from participants. The data collected was confidential and accessible only to the research team.

Statistical analysis

Data analysis was done using IBM SPSS Statistics for Windows, Version 23.0 (IBM Corp., Armonk, NY, USA). Descriptive statistics were employed and involved numbers, percentages, mean, and standard deviation (SD). Normality tests were carried out using the Shapiro-Wilk test and Kolmogorov-Smirnov test. Mann-Whitney U test and Kruskal-Wallis test were utilized for comparative analyses. A p- p-value of < 0.05 was taken as significant.

## Results

In total, 317 doctors participated, and the majority of them were aged between 20-30 years (63.5%, n = 201). There was a slight female predominance (53.9%, n = 171). Having involved interns, postgraduates, and staff from all medical specialties, roughly equal responses were recorded from the respective groups. Most doctors had one to five years of experience (64.0%, n = 203), with 22.1% (n = 70) having more than 10 years of experience. Interns who rotate through all departments were not classified under any one department (32.8%, n = 104), followed by staff and post graduates (PGs) in General Medicine (12.6%, n = 40), General Surgery (8.2%, n = 26), Obstetrics & Gynecology (7.6%, n = 24), Pediatrics (3.5%, n = 11) and other departments. Three-quarters of the responding doctors (75.4%, n = 239) did not have children. All the demographic variables are mentioned in Table 1.

**Table 1:** Demographic variables of the participants (n = 317) Data has been represented as number (N) and percentage (%) * Interns rotate through all departments and therefore were not included under any one department ** Other departments include Dermatology, ENT, Radiodiagnosis, Community Medicine, Ophthalmology, Orthopedics, Emergency Medicine, Anatomy, Forensic Medicine, Physiology, Biochemistry, Pharmacology, Microbiology, Pathology, and Dentistry

Demographic variables	Category	N (%)
Age group of respondent	20-30	201 (63.5%)
	30-40	74 (23.3%)
	40-50	28 (8.8%)
	>50	14 (4.4%)
Gender	Female	171 (53.9%)
	Male	146 (46.1%)
Designation	Intern	103 (32.5%)
	Postgraduate	96 (30.3%)
	Staff	118 (37.2%)
Department	Pediatrics	11 (3.5%)
	General Medicine	40 (12.6%)
	Psychiatry	15 (4.7%)
	Obstetrics & Gynecology	24 (7.6%)
	Interns* (rotational)	104 (32.8%)
	General Surgery	26 (8.2%)
	Anesthesia	21 (6.6%)
	Others**	76 (24.0%)
Years of experience (including internship)	1-5	203 (64.0%)
	6-10	44 (13.9%)
	>10	70 (22.1%)
Do you have children?	Yes	78 (24.6%)
	No	239 (75.4%)

The association of the various sociodemographic variables with knowledge and attitude scores is shown in Table 2.

**Table 2:** Comparison of doctor’s sociodemographic variables with knowledge and attitude scores Data has been represented in the form of Mean ± SD H = Kruskal-Wallis H test, utilized to compare more than two independent groups Z = Mann-Whitney U test, utilized to compare two independent groups * P-value < 0.05 is considered significant

Variable	Category	Knowledge score (Mean ± SD)	Attitude score (Mean ± SD)	Test statistic	p-value
Age group	20–30 years	9.96 ± 1.64	4.15 ± 1.06	H (knowledge) = 10.284	0.016*
	30–40 years	9.86 ± 1.74	4.28 ± 1.14	H (attitude) = 3.364	0.338
	40–50 years	10.92 ± 1.53	4.57 ± 1.16	-	-
	>50 years	10.28 ± 1.32	4.35 ± 1.27	-	-
Gender	Female	10.34 ± 1.51	4.18 ± 1.05	Z (knowledge) = -3.230	0.001*
	Male	9.68 ± 1.77	4.29 ± 1.15	Z (attitude) = -0.836	0.403
Designation	Intern	9.84 ± 1.74	4.01 ± 1.03	H (knowledge) = 1.417	0.492
	Postgraduate	10.09 ± 1.56	4.29 ± 1.08	H (attitude) = 5.927	0.051
	Staff	10.16 ± 1.66	4.37 ± 1.15	-	-
Department	Pediatrics	10.29 ± 1.35	4.58 ± 1.12	H (knowledge) = 5.308	0.505
	General Medicine	9.88 ± 1.75	4.26 ± 1.03	H (attitude) = 5.143	0.526
	Psychiatry	9.55 ± 1.42	4.05 ± 0.99	-	-
	Obstetrics & Gynecology	10.07 ± 1.71	4.53 ± 1.27	-	-
	Interns (rotational)	10.26 ± 1.47	4.17 ± 1.04	-	-
	General Surgery	9.76 ± 1.47	4.59 ± 0.88	-	-
	Anesthesia	10.38 ± 1.49	4.19 ± 1.24	-	-
	Others	9.74 ± 1.95	4.17 ± 1.14	-	-
Years of experience	1–5 years	9.88 ± 1.73	4.16 ± 1.07	H (knowledge) = 7.752	0.021*
	6–10 years	9.97 ± 1.33	4.44 ± 1.09	H (attitude) = 2.704	0.258
	>10 years	10.51 ± 1.54	4.30 ± 1.17	-	-
Do you have children	Yes	10.29 ± 1.46	4.24 ± 1.19	Z (knowledge) = -1.408	0.159
	No	9.95 ± 1.71	4.23 ± 1.07	Z (attitude) = -0.091	0.927

Knowledge related to child abuse

The overall knowledge was adequate (Mean = 10.03, SD 1.664). Most of the respondents (57.1%, n = 181) had adequate knowledge, with 40.1% (n = 127) possessing good knowledge, and 2.8% (n = 9) of respondents with poor knowledge scores. There was a significant association between age groups and knowledge levels. Respondents in the older age group had significantly better knowledge levels (p = .002). Female doctors had significantly higher knowledge scores than male doctors (p = 0.001). Respondents with more experience (>10 years) had significantly better knowledge scores compared to doctors with less experience (p = 0.007). The doctor's designation and department, alongside whether they had children or not, did not play a role in influencing knowledge levels (p > 0.05). The frequency of answers to knowledge questions is shown in Table 3.

**Table 3:** Respondents answers to knowledge questions (n = 317) Data has been represented as number (N) and percentages (%)

Knowledge questions	Correct response	Incorrect response
	N (%)	N (%)
Children who experience abuse typically disclose it to someone shortly after the incident	210 (66.2%)	107 (33.8%)
When a child openly declares that an adult has caused harm, the accusation must be addressed promptly	302 (95.3%)	15 (4.7%)
Child abuse is predominantly linked to the challenges of poverty	117 (36.9%)	200 (63.1%)
In a majority of cases, the abuser is a person known to the child	287 (90.5%)	30 (9.5%)
Children who are between 5-12 years of age are more susceptible to abuse	261 (82.3%)	56 (17.7%)
Does the sex of the child play an important role in the risk for abuse?	75 (23.7%)	242 (76.3%)
Is there a higher risk of abuse among children in broken families	288 (90.9%)	29 (9.1%)
Fundoscopic eye examination is essential in cases of suspected shaken baby syndrome?	251 (79.2%)	66 (20.8%)
Do children with special needs have a higher risk for abuse?	263 (83.0%)	54 (17.0%)
Most cases of child abuse are diagnosed based on examination findings alone?	160 (50.5%)	157 (49.5%)
Are unusual bruising/bone fractures with no underlying cause a suspicion for child abuse?	294 (92.7%)	23 (7.3%)
Is it mandatory to report a case of child sexual abuse under the POCSO (Protection of Children from Sexual Offences) Act?	309 (97.5%)	8 (2.5%)
What is the toll-free number to report a case of suspected child abuse? (CHILDLINE)	124 (39.1%)	193 (60.9%)
Is the POCSO (Protection of Children from Sexual Offences) Act a gender-neutral law?	241 (76.0%)	76 (24.0%)

However, certain aspects of the respondent's knowledge were severely inadequate. Only 36.9% (n = 117) of respondents agreed that child abuse was associated not only with poverty and lower socioeconomic classes, but it could also affect children from affluent backgrounds. Similarly, a small minority, 23.7% (n = 75) of respondents were aware that child abuse was equally applicable to both genders. 49.5% (n = 157) of respondents believed that child abuse was diagnosed on examination findings alone, which is not accurate. Knowledge of the toll-free number to report suspected cases of abuse was poor, with only 39.1% (n = 124) of respondents aware of the number.

Attitude toward abuse

The attitude of respondents toward child abuse was overall moderate (Mean = 4.23, SD 1.103). Mean scores for each of the designations across interns, postgraduates, and staff were 4.01 (SD = 1.03), 4.29 (SD = 1.08), and 4.37 (SD = 1.10), respectively. No significant differences across designations (p >0.05). Other sociodemographic variables, including age, gender, designation, years of experience, and having children, did not impact respondents' attitudes (p > 0.05). The frequency of correct answers to the attitude questions is shown in Table 4.

**Table 4:** Surveyors responses to attitude questions (n = 317) Data has been represented as number (N) and percentages (%)

Question	Correct response N (%)	Incorrect response N (%)
In a suspected case of child abuse, do you directly blame the parents without any evidence?	301 (95.0%)	16 (5.0%)
Do you have any awareness regarding the Indian law pertaining to child abuse?	164 (51.7%)	153 (48.3%)
Do you think it is the physician's responsibility to ensure mental and physical well-being of a confirmed case of child abuse?	294 (93.3%)	21 (6.7%)
It is important to raise awareness and knowledge levels regarding child abuse among the medical fraternity?	316 (99.7%)	1 (0.3%)
Do you feel that you have adequate knowledge related to child abuse?	80 (25.2%)	237 (74.8%)
Are you confident that you will be able to detect and report a case of child abuse with your current knowledge?	186 (58.7%)	131 (41.3%)

**Figure 1:**
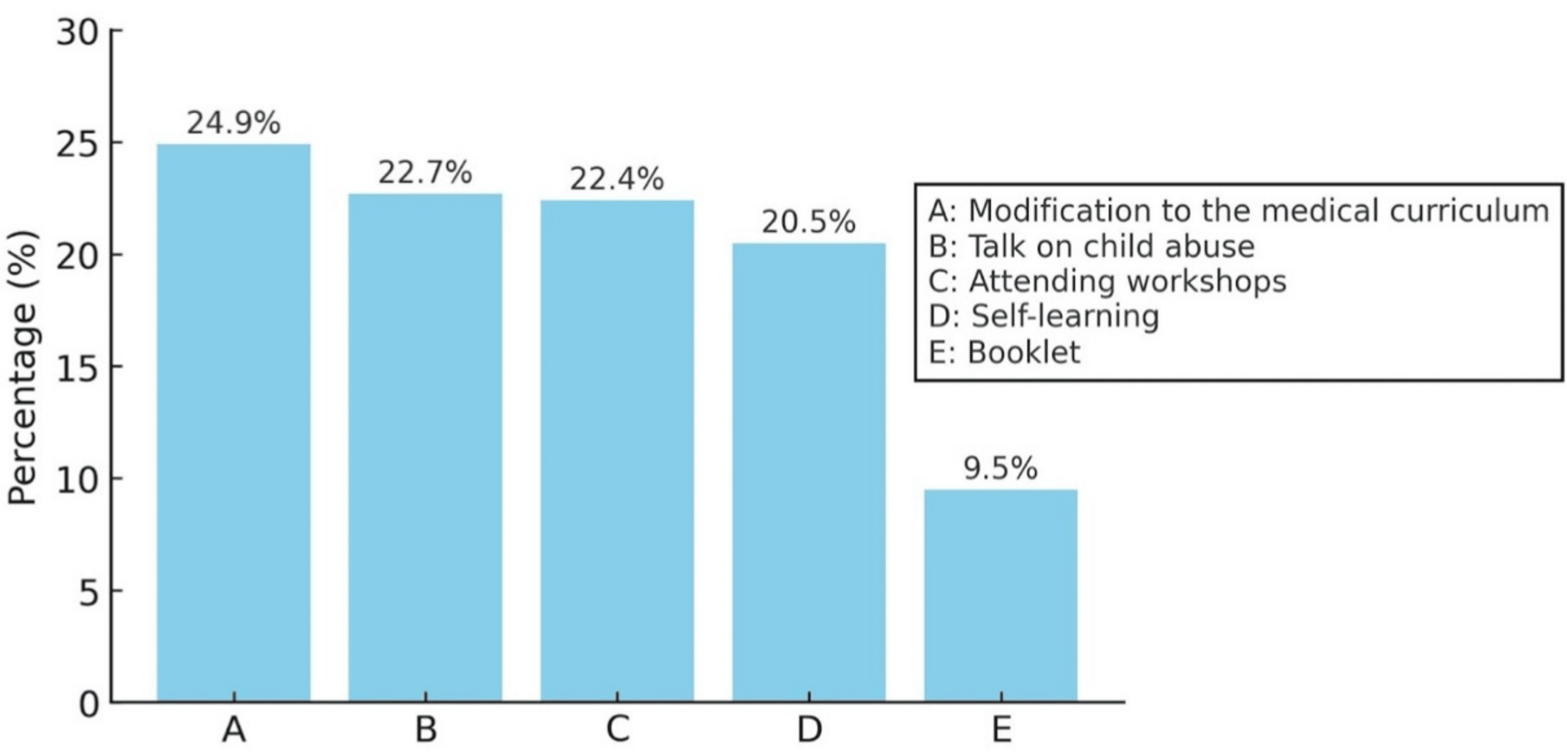
How respondents prefer to enhance their knowledge related to child abuse Data has been represented in percentage (%)

On being asked through what means respondents would like to improve their knowledge related to child abuse, most doctors suggested changes to the medical curriculum, followed by attending talks and workshops on the subject. The responses have been illustrated in Figure 1.

Practices toward abuse

Most doctors (58.7%, n = 186) answered two out of the three questions correctly, whereas only 131 respondents (41.3%) answered all three questions correctly. The majority (84.5%, n = 268) of doctors correctly identified the first step in reporting abuse. However, only about half (50.2%, n = 159) correctly answered that cases of abuse need not be referred to government hospitals for further treatment. A total of 317 respondents participated in the survey addressing whether they had encountered a case of child abuse and, if so, whether it was reported. The majority of respondents (75.7%, n = 240 ) indicated that they had not encountered a case of child abuse. Among the respondents who encountered a case of child abuse, 71.4% (n = 55) reported the incident, while the remaining did not, demonstrating poor reporting and intervention practices. Correct responses to the practice questions are shown in Table 5.

**Table 5:** Surveyors response to practice questions (n = 317) Data has been represented as number (N) and percentages (%)

Question	Correct response N (%)	Incorrect response N (%)
In the hospital setting, what is the first step in reporting a case of suspected child abuse?	268 (84.5%)	49 (15.5%)
Can a private medical practitioner report a case of suspected child abuse?	308 (97.2%)	9 (2.8%)
Do all suspected/confirmed cases of child abuse need to be referred to government hospitals for further management?	159 (50.2%)	158 (49.8%)

**Figure 2:**
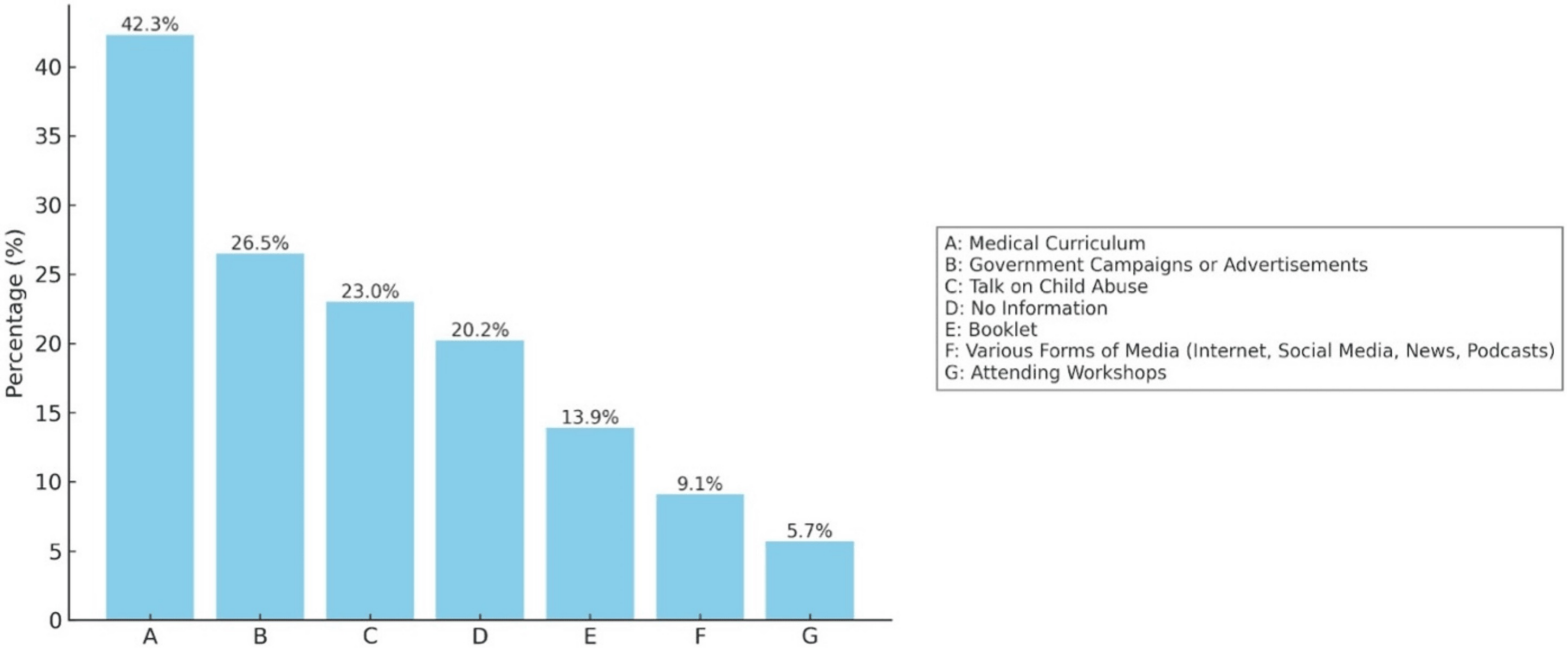
How respondents obtained information on child abuse (multiple responses) Data has been represented in percentage (%)

Most respondents attained their information on child abuse through the medical curriculum, followed by governmental campaigns. Further details on respondents' source of information are illustrated in Figure 2.

**Figure 3:**
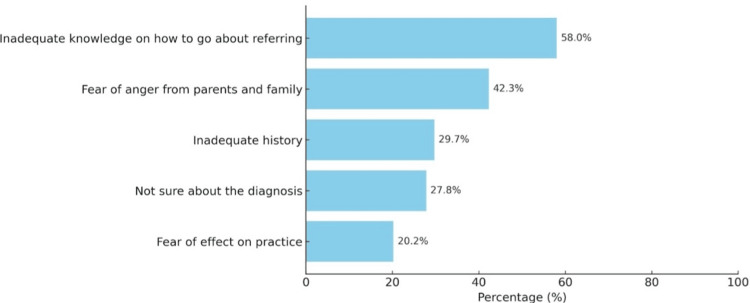
Barriers toward reporting cases of child abuse (multiple responses) Data has been represented in percentage (%)

The major hurdle, as reported by the doctors toward reporting cases of child abuse, was a lack of knowledge of the referral process, followed by fear of anger from parents and family. Other perceived obstacles stated by surveyors toward reporting cases of child abuse are illustrated in Figure 3.

Spearman’s rho correlation demonstrated a weak but significant positive correlation between knowledge and attitude (r = 0.130, p = 0.015).

## Discussion

This was a cross-sectional study of the knowledge, attitudes, and practices of doctors regarding child abuse in the region of Mangalore, Karnataka, India. It highlighted that doctors in this region have adequate knowledge and a moderate attitude toward child abuse, while there are significant gaps in their practice. Previous studies have similarly reported knowledge-practice discrepancies among medical professionals. This could be due to the fact that despite adequate levels of knowledge, doctors were unable to translate this knowledge into effective practices toward assisting and reporting suspected cases of abuse.

Insights on respondents' knowledge

Child abuse remains an issue that is seldom discussed or given much significance. It can present in various forms and involves children of all ages and from various backgrounds. Therefore, possessing relevant knowledge on the issue is vital in detecting cases of suspected abuse. 57.1% (n = 181) of doctors in our study had adequate knowledge. However, a subset (40.1%, n = 127) of the respondents had good knowledge, indicating that it was a knowledgeable group overall. Various studies have revealed a similar level of adequate knowledge [8-11]. However, on the other side of the spectrum, studies have revealed poor levels of knowledge. In our close vicinity, a study conducted in Bagalkot district (Karnataka, India) by Kirankumar et al. revealed low levels of knowledge, differing from our findings [7]. Similarly, in Gujarat, researchers found a lack of knowledge related to child abuse, especially in relation to physical and social indicators of abuse [6]. This shows that there are differences in knowledge levels among medical professionals working across various regions within the same country. Significant variations in medical training, local policies, and institutional emphasis are some of the reasons contributing to these variations.

Our study revealed that respondents in the older age group (40-50 years; 8.8%, n = 28) had significantly higher knowledge scores than those who were younger (p = 0.002). This difference in knowledge levels by age was also noted in a study involving physicians and interns, where older respondents had better knowledge levels [12]. This difference could arise from the fact that with age comes maturity and hence a better understanding of the potential factors that could play a role in child abuse. Amongst the respondents, female doctors were more knowledgeable than their male counterparts in our study (p = 0.001). In a study by Al-Moosa et al., female pediatricians were more likely to pick up on cases of perceived maltreatment [13]. This gender difference could be attributed to traditionally greater involvement of women in child upbringing, possibly making them more empathetic and aware of the circumstances surrounding child abuse. However, other studies did not portray the same picture, with no gender variations in knowledge present [8,11-12]. A number of factors could play a role in giving rise to such variations, including differences in medical education and training-particularly around reporting protocols-variations in clinical practice, and perhaps most importantly, cultural differences among nations.

In a study conducted among medical interns, residents, and staff in Al Qassim, Saudi Arabia found that doctors with children had better knowledge scores than those who did not [12]. However, our survey did not show any significant differences in knowledge between doctors with and without children (p = 0.159). This could be due to knowledge being primarily obtained through the medical curriculum, with no form of additional community/social awareness for those respondents who are parents. Just as older respondents had better knowledge scores, those with >10 years of experience had significantly better knowledge scores than those with one to five years of experience (p = 0.007). Experience counts, and this was demonstrated in the above results. On a similar note, a survey by Alkathiri et al. revealed that experienced respondents were more knowledgeable about reporting measures compared to their less experienced colleagues [14].

Despite years of experience contributing to a better level of knowledge, the surveyors' designation did not have a significant impact on knowledge scores (p = 0.432). Adding weight to our findings is a study conducted in Iran, among 130 pediatric physicians and nurses [8]. There was no significant difference in the level of knowledge among residents and staff of the pediatric department, including nurses [8]. Similarly, there was no significant difference in the level of knowledge in a study involving physicians and interns [12]. Training, therefore, needs to be imparted to doctors practicing at all levels to ensure there exists a low threshold for suspecting cases of abuse. Interdepartmental differences in knowledge levels in our survey were not significant (p > 0.05). This could arise due to a lack of focused postgraduate training offered, especially for those involved in direct care of children, such as in pediatrics, general medicine, and emergency medicine. Surprisingly, a study by Aldukhayel et al. revealed that pediatricians had lower knowledge scores than those belonging to other specialties [12].

In relation to legal aspects, the overwhelming majority (97.5%, n = 309) of doctors in our survey knew of the mandatory requirement to report cases of abuse. The respondents, therefore, were aware of their legal responsibilities, which is an essential component in the path to improving the rate of underreporting. One contributing factor could be the rising public awareness and efforts by the judiciary, including a special law implemented in 2012 by the Government of India in the form of the Protection of Children from Sexual Offences (POCSO) Act [15]. This degree of awareness of legal obligation was not evident in studies from other regions. More than 80% of respondents were not aware of their legal obligation to report cases in a study done in Kuwait [13]. Despite knowledge of the legal aspects, our survey showed that doctors were unaware that abuse could affect male children equally. Studies done in India have revealed that both genders are equally susceptible to abuse [2,16]. This lack of knowledge pertaining to abuse in the male child could be detrimental to detecting and reporting cases of male child abuse. On the other hand, an overwhelming majority of doctors (90.5%, n = 287) were aware that the perpetrator in cases of abuse was likely someone the child knew. A survey by the Indian government showed that the majority of abusers were people whom the child knew either in a personal or professional capacity [2].

Identifying potential cases of abuse can be challenging at times, especially since less than 5% of victims present with physical signs [17]. Therefore, a thorough history becomes essential in screening for abuse. Our study showed that only half the respondents (50.5%, n = 160) agreed that diagnosing a case of abuse would not depend on examination findings alone. This highlights gaps in existing knowledge, which would potentially diminish systems of identification and reporting. This deficiency could also be a reason why there is underreporting, especially among healthcare systems. Additionally, children from various economic backgrounds have been known to be victims of abuse [18]. Half the doctors (50.5%, n = 160) answered that children who are abused predominantly belong to the lower socioeconomic class. However, this is not the case, and healthcare professionals must keep a low threshold for suspecting abuse in children, even from affluent backgrounds.

Attitude related to child abuse

Doctors demonstrated moderate attitudes toward child abuse in our study. A study on physicians and nurses revealed excellent attitudes in 64.2% of the participants [14]. Positive attitudes among doctors were also revealed in another study [12]. Most (95%, n = 301) of the doctors in our study stated that it was wrong to blame the parents in a suspected case of abuse without any evidence. The topic of abuse is considered taboo and hence has severe implications for both the child and the family involved. Therefore, it is essential to develop systems to investigate and provide both physical and psychological support to victims and their families. 

An overwhelming majority (93.3%, n = 294) of doctors felt it was their responsibility to ensure the mental and physical well-being of a victim of abuse. This demonstrates their eagerness to fulfill their medical and legal duties despite the obstacles faced in managing such cases. According to a study in northern France, a predominant number (94%) of family physicians felt they were personally involved in the care of children who were at risk of abuse [9]. These encouraging findings have been reproduced in various other studies. In Bagalkot district of Karnataka, India, medical professionals agreed that it was their duty to protect children's health [7]. Additionally, another study from Gujarat, India, showed that only 3% of respondents stated that recognition of child abuse was not an essential part of their profession [6]. This study also showed that more than 90% of respondents sought to improve their knowledge and understanding of child abuse [6]. This is in agreement with our findings, where almost all (99.7%, n = 316) of the doctors wished to increase their knowledge and raise awareness on child abuse, thus demonstrating positive attitudes toward the issue. Likewise, a majority (95.5%) of healthcare staff in a study done in Iran were in support of educational programs to further their knowledge related to child abuse [8].

Modifications to the medical curriculum were the most preferred way of improving knowledge, with close to 25% (n = 79) of respondents looking for changes to the curriculum. Attending talks and workshops was a roughly equally attractive choice for doctors to gain more knowledge.

Practice related to child abuse

The practice of doctors in managing and reporting abuse was unsatisfactory. Specific protocols on the identification and reporting of child abuse are not widely implemented across medical colleges and hospitals. In spite of this, doctors bear both professional and legal responsibility in detecting but also mandatorily reporting cases of abuse. According to our survey, 75.7% (n = 240) of doctors did not encounter a case of abuse in their practice. In contrast, a study in Kuwait found that half the doctors came across cases of abuse in the year preceding the survey [13]. This difference could be attributed to gross underestimation of the prevalence and underreporting from various institutions, including medical institutions. Among the 24.3% (n = 77) of doctors in our study who came across a case of child abuse, 28.6% (n = 22) of respondents did not report despite the law stating reporting of cases as mandatory. Inanci et al. showed that among the 21.5% of doctors in primary care settings who participated in their survey who had encountered a case of abuse, half the respondents omitted to report the case [18].

Inadequate knowledge of the referral process was the most significant barrier towards reporting cases of abuse, followed by fear of anger from parents and family. Insufficient knowledge of the reporting process stood out as a major obstacle in a number of other studies [8,14,18]. This clearly demonstrates that gaps in knowledge need to be addressed to facilitate effective surveillance and to empower medical professionals to report suspected cases. Providing treatment to suspected/confirmed cases of abuse can be done in both private and government institutions [19]. This fact needs to be reinforced to healthcare workers so as to prevent any iatrogenic delays in providing prompt medical treatment. Our study findings align with research conducted in India and globally, emphasizing the need for improved education and reporting mechanisms.

Limitations of the study

This study focused exclusively on doctors, excluding nurses and other healthcare professionals who are vital members of the multidisciplinary team. These professionals play an important role in both escalating concerns and reporting suspected cases of abuse. Their exclusion from the study means we are unaware of what barriers they particularly face in voicing their concerns regarding child abuse, as well as their knowledge, attitude, and practice toward the subject.

There is potential for sampling bias when using a convenient method of sampling. Furthermore, the study was conducted in a single urban region of one state. The results, therefore, may not be generalisable or even replicable given the wide variations in cultures, medical training, and local practices that exist in different regions of India. 

Additionally, due to the nature of the study, the potential for self-reporting bias must be taken into consideration when interpreting the study results.

Recommendations

Some of our key recommendations include the integration of child abuse training into the medical curriculum, with mandatory training during undergraduate and postgraduate education, including educating doctors about child protection laws and their obligations. This would be further supplemented by regular workshops and continued medical education (CME) programs focused on identifying and reporting child abuse. Additionally, they would also provide doctors a forum to raise concerns and empower them to translate their knowledge into meaningful interventions to report cases of abuse. Hospital-based protocols for reporting abuse should establish a streamlined mechanism to facilitate timely reporting and intervention, enabling staff to perform their duties without uncertainty or hesitation.

## Conclusions

This study highlights that doctors in this region have adequate knowledge and a moderate attitude toward child abuse, while there are significant gaps in their practice. This could be due to the fact that despite adequate levels of knowledge, doctors were unable to translate this existing knowledge into effective practices toward assisting and reporting suspected cases of abuse. Addressing these barriers through structured training and institutional protocols can improve detection and intervention strategies.

## Appendices

**Table 6:** Structured questionnaire on the demographic characteristics of participants, the knowledge, attitude, and practice related to child abuse among doctors in Mangalore, India

Question	Response options
Section A: Demographic characteristics of participants	
1. Age of respondent	20-30
	30-40
	40-50
	>50
2. Gender	Female
	Male
3. Designation	Intern
	Postgraduate
	Staff
4. Department	________ (In case of interns, kindly mention department as rotational)
5. Years of experience (including internship)	1-5
	6-10
	>10
6. Do you have children?	Yes
	No
Section B: Knowledge	
1. Children who experience abuse typically disclose it to someone shortly after the incident?	Yes
	No
	Don’t know
2. When a child openly declares that an adult has caused harm, the accusation must be addressed promptly	Yes
	No
	Don’t know
3. Child abuse is predominantly linked to the challenges of poverty	Yes
	No
	Don’t know
4. In a majority of cases, the abuser is a person known to the child	Yes
	No
	Don’t know
5. Children who are between 5-12 years of age are more susceptible to abuse	Yes
	No
	Don’t know
6. Does the sex of the child play an important role in the risk for abuse?	Female > Male
	Male > Female
	No role at all
7. Is there a higher risk of abuse among children in broken families?	Yes
	No
	Don’t know
8. Fundoscopic eye examination is essential in cases of suspected shaken baby syndrome?	Yes
	No
	Don’t know
9. Do children with special needs have a higher risk of abuse?	Yes
	No
	Don’t know
10. Most cases of child abuse are diagnosed based on examination findings alone?	Yes
	No
	Don’t know
11. Are unusual bruising/bone fractures with no underlying cause a suspicion for child abuse?	Yes
	No
	Don’t know
12. Is it mandatory to report a case of child sexual abuse under the POCSO (Protection of Children from Sexual Offences) Act?	Yes
	No
	Don’t know
13. What is the toll-free number to report a case of suspected child abuse? (CHILDLINE)	1099
	1080
	1098
	Don’t know
14. Is the POCSO (Protection of Children from Sexual Offences) Act a gender-neutral law?	Yes
	No
	Don’t know
Section C: Attitude	
1. In a suspected case of child abuse, do you directly blame the parents without any evidence?	Yes
	No
2. Do you have any awareness regarding the Indian law pertaining to child abuse?	Yes
	No
3. Do you think it is the physicians responsibility to ensure mental and physical well being of a confirmed case of child abuse?	Yes
	No
4. Is it important to raise awareness and knowledge levels regarding child abuse among the medical fraternity?	Yes
	No
5. Do you feel that you have adequate knowledge related to child abuse?	Yes
	No
6. Are you confident that you will be able to detect and report a case of child abuse with your current knowledge?	Yes
	No
7. How would you like to improve your knowledge related to child abuse? (Choose any one option)	Booklet
	Talk on child abuse
	Attending workshops
	Modification to medical curriculum
	Self-learning
Section D: Practice	
1. How have you obtained your information on child abuse? (Multiple options applicable)	Booklet
	Talk on child abuse
	Government campaigns/Ads
	Medical curriculum
	Attending workshops
	Others: ________ (Please specify)
	No information
2. Have you encountered a child abuse case?	Yes, it was reported
	Yes, but not reported
	No
3. What do you think is the reason for not reporting a case of child abuse? (Multiple options applicable)	Fear of anger from parents and family
	Inadequate knowledge of how to go about referring
	Not sure about diagnosis
	Inadequate history
	Fear of effects on practice
4. In the hospital setting, what is the first step in reporting a case of suspected child abuse? (Tick any one option)	Reporting to ASHA worker
	Informing nearest police station
	Reporting to medical superintendent of the hospital
	Not sure
5. Can a private medical practitioner report a case of suspected child abuse?	Yes
	No
6. Do all suspected/confirmed cases of child abuse need to be referred to government hospitals for further management?	Yes
	No
